# Tracheal reconstruction: mini-review and anatomical study on the use of antero lateral thigh fascial flap for heterotopical transplantation

**DOI:** 10.1007/s12055-022-01354-x

**Published:** 2022-04-04

**Authors:** Rossella Sgarzani, Giuseppe Meccariello, Giannicola Iannella, Franco Stella, Luca Negosanti

**Affiliations:** 1 Dipartimento di Medicina Specialistica, Diagnostica e Sperimentale, Università di Bologna, via Massarenti 9, Bologna, Italy; 2 U.O. Otorinolaringoiatria, Ospedale Morgagni Pierantoni, Forlì, AUSL Romagna Italy; 3 Dipartimento Organi di Senso, Università La Sapienza, Rome, Italy; 4 Servizio di Chirurgia Plastica, Montecatone Rehabilitation Institute, Imola, Italy

**Keywords:** Tracheal reconstruction, Heterotopical transplantation, Antero lateral thigh flap

## Abstract

**Background:**

The reconstructive tracheal options for extensive lesions still remain limited and although a valid substitute is required unfortunately, the biomechanical tracheal characteristics do not allow an easy replacement. In this study we reviewed the described options and investigated, in human cadaver model, whether thigh fascia can be used, as an alternative to forearm fascia, as recipient site for trachea graft heterotopical allotransplantation.

**Anatomical study:**

In three fresh cadavers, 3 tracheal graft, 6 radial forearm (RF) fascial flaps and 6 antero-lateral thigh (ALT) fascial flaps were harvested. For each flap we simulated the heteretopical transplantation of the trachea in each fascial flap, and the harvesting of the composite graft as a free flap. The composite graft was finally decomposed at bench and the pedicle was injected to confirm fascial vascularization. The main measured outcomes were: flap fascia vascularization after pedicle injection, average time of flap harvesting, number of perforators included in ALT fascial flap and diameter of the vessels for anastomosis. Difficulties were noted, in order to compare RF flap and ALT flap.

**Results:**

Fascia vascularization was confirmed in all cases by pedicle injection. The main difficulty with radial flap was to harvest the fascial layer due to its thinness and its strong adherence to palmaris longus tendon, while the main difficulty with ALT flap was to prevent any traction on the perforators. The average time of flap harvesting and graft inset (by a junior plastic surgeon) was 1 h and 30 min for radial forearm flap and 2 h and 10 min for ALT flap.

**Conclusion:**

Despite many different techniques proposed in the literature, tracheal heterotopical allotransplantation still seemed the most promising, and ALT flap promised be a feasible alternative for heterotopical transplantation of trachea.

## Introduction

The reconstructive options of trachea still remain limited. Usually minor defects, less than half in adults and less than one third in children, can be treated with segmental resection and anastomosis, while extensive lesions need substitute tissues [1, 2].

Tracheal allotransplantation was successfully described by Delaere in 6 cases [3]. Since the trachea lacks an identifiable vascular pedicle, the technique applied a reconstructive method called prelamination, first described in 1994 by Pribaz and Fine [4], and often used for total nasal or ear reconstruction. According to Delaere’s procedure, firstly the donor trachea is heterotopically transplanted in a radial forearm (RF) fascial flap, and then the composite free flap is transferred, as a vascular unit to replace the tracheal defect, after waiting for at least three months in order to determine the revascularization of the graft from surrounding tissues [3].

Our report investigates the preliminary results of a possible alternative technique for vascularized tracheal allotransplantation. Thus, human cadaver models were used to evaluate whether antero-lateral thigh (ALT) fascia could be used, instead of RF fascia, as recipient site for trachea graft in the heterotopical allotransplantation framework.

## Evolution of operative techniques

The ideal substitute should have particular features: 1) resistent to dislocation and stenosis; 2) flexible longitudinally, 3) limiting the accumulation of secretions and bacteria [1].

Grillo analyzed and classified various tracheal substitutes into four groups: 1) foreign materials, 2) tissue engineered parts, 3) autologous tissues, 4) tracheal transplantation [5].

Different foreign materials have been used in animal models to replace the trachea, such as titanium mesh, polyurethane and polytetrafluoroethylene (PTFE), a non-biodegradable polymer used widely in reconstructive vascular surgery [6–8]. Unfortunately, complications have been registered such as emigration, infection and extrusion due to the use of these non-resorbable materials.

Tissue engineering attempted to find an organic solution to reliably replace the trachea [9–11]. Recently, Scierski implanted a cylindrical tracheal implant, made of polymers containing various forms of carbon fibers, impregnated with polysulfone (PSU), in 10 ovines [12]. After 24 weeks it was found that vascularized connective tissue covered the inner surfaces, whilst another type of tissue, which resembled histological structure of normal tracheal wall, covered the outer surfaces.

Autologous tissues have been used as grafts and as flaps; multiple tissues were used as grafts, such as bronchial patches, dermis, pericardium, and aortic grafts. In 2000, Martinod implanted a thoracic aortic patch with temporary stenting, achieving metaplasia of the aortic tissue, and introducing a new era of in situ regeneration for tissue engineered trachea replacement [13].

Prelamination method, first described in 1994 by Pribaz and Fine [4], was applied to tracheal reconstruction. This was a staged procedure in which additional tissue (a cartilage graft) was shaped and added to an existing flap (radial flap or omentum flap), and, after revascularization of the graft from surrounding tissues (about 3 weeks), the composite free flap was transferred as a vascular unit to replace the tracheal defect [2].

The donor trachea was used to replace the original trachea as a non-vascularized graft, without encouraging results. In fact, mucosa was identified as the main antigenic source by Beigel et al. [14] and Bujia et al. [15]. Experimental trials involving de-epithelization, merthiolate treatment and cryopreservation to inhibit immunogenicity, while maintaining structural integrity, resulted in failures. Although rejection was avoided, the failure has been due to fibrosis, stenosis or necrosis [2].

The vascular supply to trachea has different sources from the thyroid, esophagus, pharynx, larynx and other surrounding tissue, this pattern does not allow performing a standard vascularized organ transplantation. In a relatively recent study on swine model [16], the trachea was harvested with the surrounding organs and heterotopically transplanted as a visceral compartment supplied from branches of the carotid artery and internal jugular vein. The animals were sacrificed after 14 days and the graft appeared well vascularized and healthy.

Nowadays, investigators mainly focused on indirect revascularization of donor trachea by prelamination. For these purposes, donor trachea was wrapped in a soft tissue flap, after revascularization of the graft from surrounding tissues (about 3 weeks), the prelaminated composite free flap was transferred [3, 17]. These methods have produced encouraging results, but they were time-consuming processes due to the staged nature of the grafting.

Xu et al. [17] used the greater omentum as a tracheal transplantation carrier in 3 patients. The main disadvantage of the procedure was the need for abdominal surgery.

Delaere et al. [3] reported 6 successful transplantations with a three-step procedure:

firstly, a heterotopical transplantation of the donor trachea in the recipient forearm fascial flap was done with concomitant administration of immunosuppression therapy; secondly, after 3 months, the graft was revascularized from surrounding tissues and donor respiratory mucosa was completely regenerated, then donor respiratory epithelium was replaced with recipient buccal mucosa graft. Finally, after ingrowth of the recipient mucosal graft, the orthotopic transplantation of the composite graft as a free flap could be performed while the immunosuppression therapy was gradually stopped.

The main advantage of this procedure was the limited administration of immunosuppression therapy compared to the other composite tissue allotransplantations (CTA). In fact, Delaere described [18] the withdrawal of immunosuppression in a safe manner and some tricks such as the second procedure of removal of donor respiratory epithelium and replacement with recipient buccal mucosa graft. These allowed all immunogenic non-cartilagineous tissues to be gradually replaced by cells from recipient mucosa, the only permanent donor tissue was the cartilage, not causing allorejection.

At present, indirect revascularization of donor trachea seemed to be the best clinical option.

## Anatomical study

The study was conducted at the Anatomy Laboratory of the “Centre Hospitalier Universitaire” in Liege, Belgium, three fresh cadavers were used.

In each cadaver a tracheal segment, composed by 10 to 12 rings, was harvested. Then two RF fascial flaps (6 in total) and two ALT fascial flaps (6 in total) were harvested by the same junior plastic surgeon.

All trachea grafts were healthy and not calcified, each graft was used to simulate heterotopical transplantation 4 times, for the 4 flaps of the cadaver.

For each flap, we simulated the heterotopical transplantation of the trachea graft in the fascial flap, then the composite graft as a free flap was harvested. The composite graft was finally decomposed at bench and the pedicle was injected.

Fascial vascularization after pedicle injection, average time of flap harvesting, number of perforators included in ALT fascial flap, diameter of the vessels for anastomosis and notes on the difficulties were collected.

## Harvesting technique

**To harvest the tracheal graft,** a skin incision was made between the two mastoid processes, passing above the jugular notch. A flap of skin and platysma was elevated to the lower border of the mandible. Dissection of sternohyoid and sternothyroid muscles exposed the laryngo-tracheal complex. Trachea was then separated from esophagus posteriorly and thyroid anteriorly. A 10 rings segment in 1 cadaver and 12 rings segments in 2 cadavers were harvested. The trachea was then treated at bench, removing the membranous part and the anterior pre-tracheal fascia. The inner mucosa was left attached.

**The RF fascial flap** was harvested as described by Delaere [3]. Trachea was then positioned on the forearm and wrapped with the fascia. Trachea was fixed at both margins to the skin with sutures to maintain its length. Skin was then sutured to tracheal edges.

To simulate the **harvest of the composite graft** a random rectangular skin flap, as long as the graft and wide as the posterior tracheal wall, to reconstruct was drawn laterally or medially to one edge of the composite graft. The flap was elevated, rotated in order to reconstruct the tracheal posterior wall and sutured to the other edge. The composite flap was then dissected within radial pedicle.

**Fig. 1:**
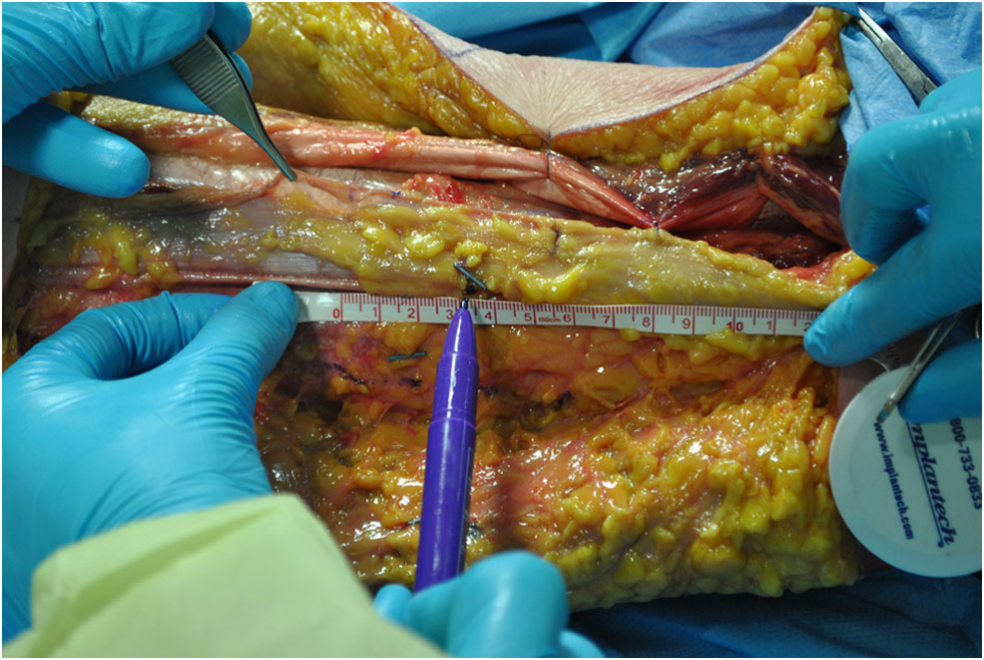
The fascia was incised on the medial aspect of the thigh maintaining all perforators. The pedicle was identified inside the septum, confirming the origin of the perforator from it. The fascial flap was centered on the perforator, considering trachea graft dimensions

**Fig. 2:**
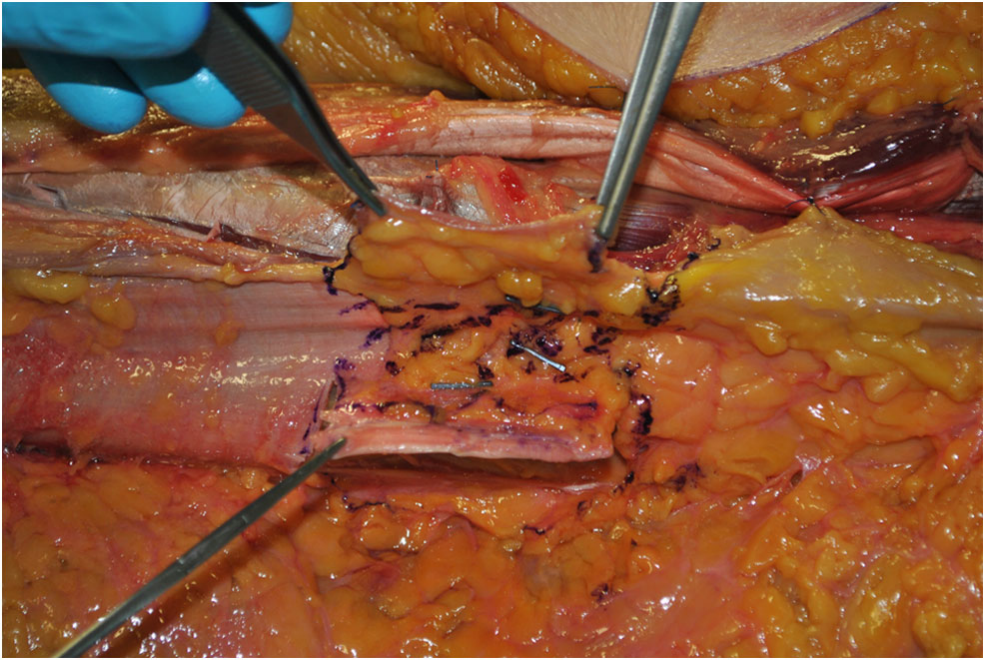
The vertical edges of the fascia were incised entirely, but not the horizontal ones, to avoid any possible tension on the vessels

**Fig. 3:**
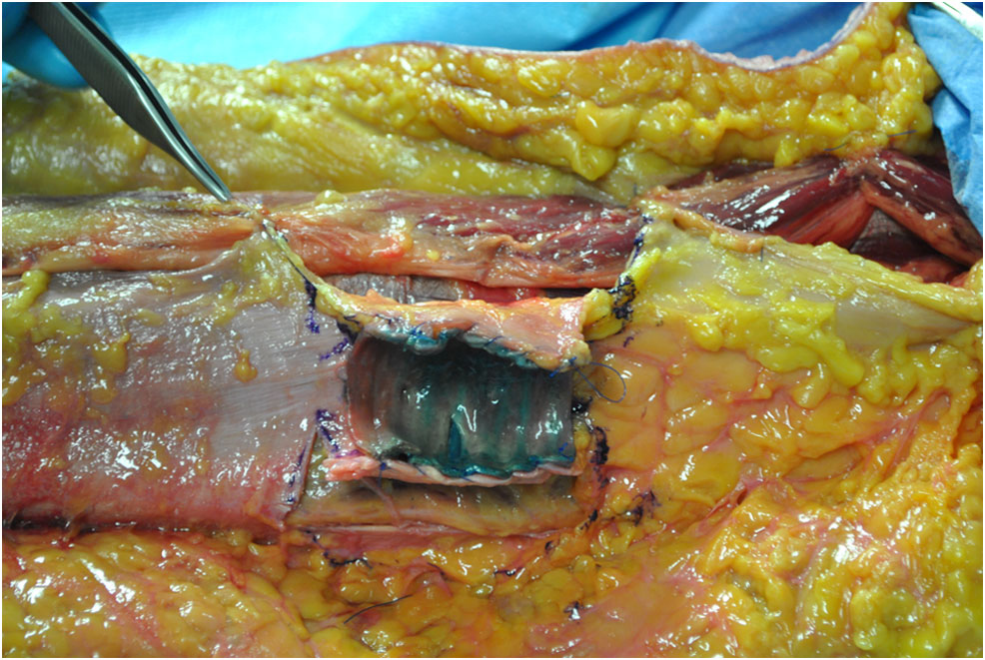
Fascia was wrapped around the trachea and the vertical margins of the fascia flap were then sutured to tracheal edges

**Fig. 4:**
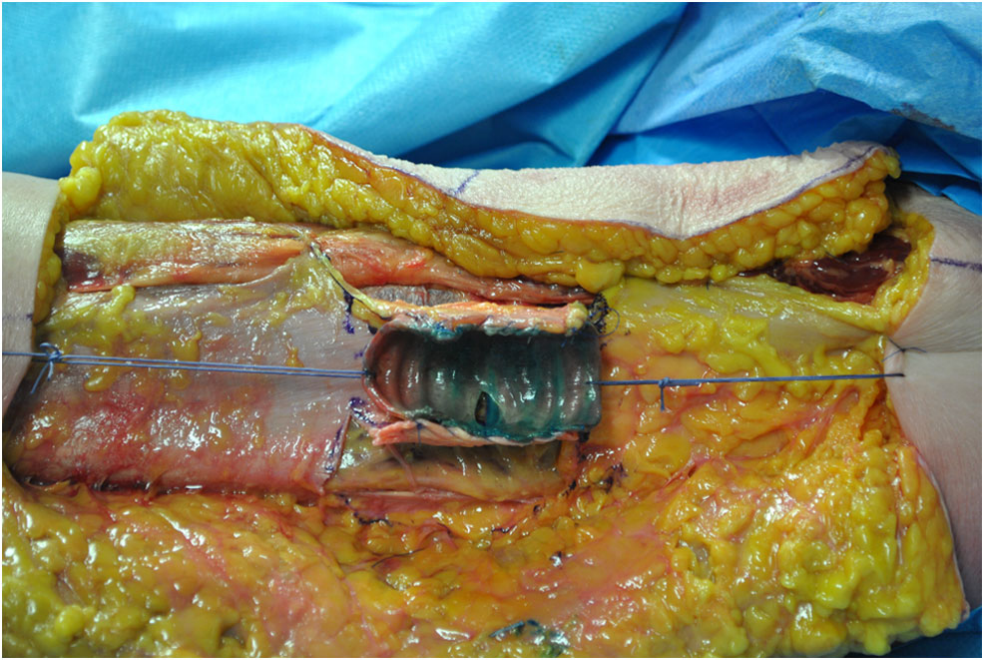
Superior and inferior borders of the trachea were fixed to the skin with sutures, to maintain the graft length

**Fig. 5:**
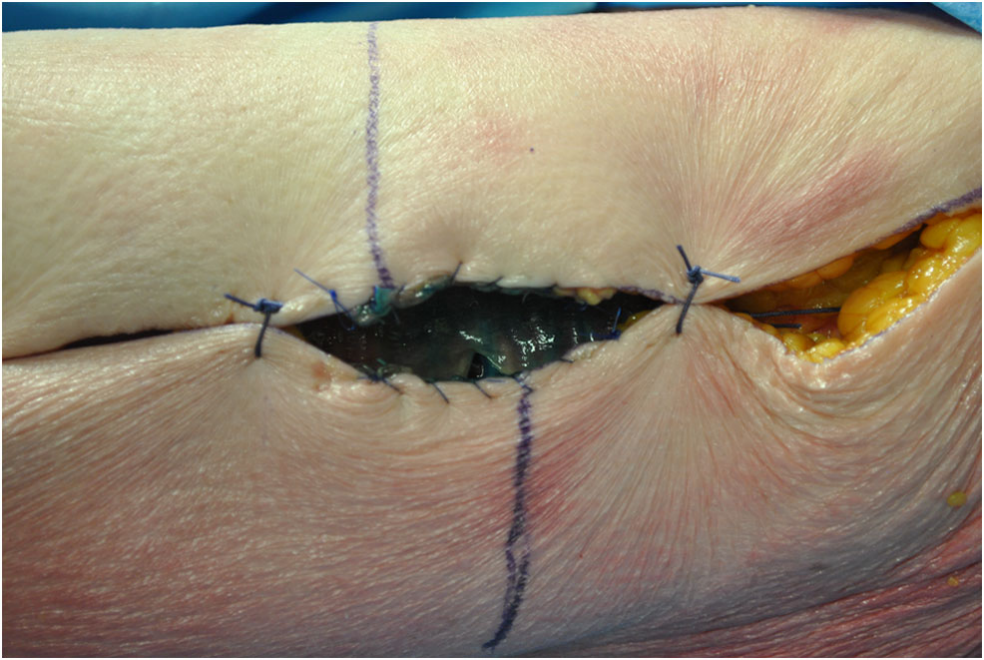
The margins of skin incision were then sutured to trachea and thigh incision was sutured

**ALT fascial flap** was harvested with a similar technique. A skin incision was made medially to the cutaneous projection of the intermuscular septum, between rectus femoralis and vastus lateralis muscles, described by a line from the anterior superior iliac spine to the patella lateral margin. The incision was made through the skin and subcutaneous tissue until the fascia; supra-fascial dissection was then performed. All perforators were identified on the supra-fascial layer and ligated distally, this made the later identification in the sub-fascial layer very easy. An incision was made through the fascia on the medial aspect of the thigh and the dissection continued laterally in the subfascial plane, preserving all possible perforators. The intermuscular septum between vastus lateralis and rectus femoralis muscles was then opened to identify the descending branch of the lateral circumflex femoral artery and the origin of the perforators. The best possible perforator was then selected and marked. The fascia was incised in a rectangular shape, centered on the selected perforator; the dimension was set on that of the tracheal graft (Fig. 1). The lateral edges of the fascia were entirely incised, but not the cranial and caudal ones to avoid any possible tension on the vessels (Fig. 2). The fascial flap was wrapped around the trachea and the lateral margins were then sutured to tracheal edges (Fig. 3). Superior and inferior boarders of the trachea were also fixed to the skin with sutures, to maintain the graft length (Fig. 4). The margins of skin incision were then sutured to trachea in order to obtain the same result of forearm in setting (Fig. 5) and thigh incision was sutured.

To simulate the **harvest of the composite graft**, a random rectangular skin flap, as long as the graft and wide as the posterior tracheal wall, to reconstruct was drawn laterally or medially to one edge of the composite graft. The flap was elevated, rotated in order to reconstruct the tracheal posterior wall and sutured to the other edge.

The fascia was then completely detached and the septum was opened to isolate the vascular pedicle until its origin.

## Our experience

In all cases we obtained a composite graft of trachea and fascia and fascia vascularization was confirmed by pedicle injection.

The main problem encountered during RF dissection was the difficulty to harvest the fascial flap due to the thinness of the fascia and its strong adherence to palmaris longus tendon. However, ALT fascia was clearly thicker and easier to dissect, while the main problem encountered during ALT flap dissection was to prevent any traction on the perforators.

In 2 ALT flaps we found 2 perforators on the same vertical axis, and preserved them both.

The average time of flap harvesting and graft inset was 1 h and 30 min for radial forearm flap and 2 h and 10 min for ALT flap.

The average diameter of the artery for anastomosis was 2.5 mm (range 2 to 3 mm) for ALT flap and 3 mm (range: 2.0 to 4.0 mm) for RF flap. In 3 of the 6 ALT flaps, the 2 venae comitantes joined in one larger vein, the average diameter of the vein for anastomosis was 2 mm (range 2 to 3.5 mm) for ALT flap and 1.7 mm (range: 1.5 to 2 mm) for RF flap.

## Discussion

The aim of our work was to prove the technical feasibility of preparing the composite graft using the ALT fascial flap as a carrier, instead of RF fascial flap.

The thinness of RF fascia and its strong adherence with the palmaris longus tendon determine a difficult dissection. This issue might be partly solved by harvesting the fascial flap quite distant from the elbow, in order to find the muscular part of the palmaris longus instead of its tendinous part.

ALT fascia flap was harvested easily in all reported cases. Fascia is very strong and easy to dissect, due to its low adherence to subcutaneous and muscular planes. The pedicle could be only visualized inside the intermuscular septum, without dissecting it, in order to reduce vascular damage due to tension on the pedicle. Leaving the fascia partially attached on the axis of the perforators allowed reduction of tension on the perforators, while the sutures between trachea and skin reduced the tension on the vascular pedicle, maintaining the own length. The well-known problem linked to vascular anatomy of variability in ALT flap could be solved by preoperative angio-tomography study. Graft harvesting was quite simple and harvesting time difference between the two methods (average 40 min) was acceptable. In both methods the pedicle was long enough to safely perform.

anastomoses to neck vessels without tension, furthermore the vessels caliber were adequate in all flaps.

The use of ALT flap in-vivo could be a solution to the well-known drawbacks of the RF donor site - sacrifice of a major artery of the limb, the unpleasant visible scar in the forearm region and possible scar related symptoms such as pain and numbness [19]. The comparison between these two flaps and the superiority of perforator flaps about donor site morbidity is well known [20]. Moreover, according to Delaere’s procedure, the patient will have the heterotopic trachea allograft in place for at least three months, before its transfer, and we would like to highlight that a wound and a dressing on the thigh is much easier to live with and less disturbing, than on the forearm.

## Conclusion

Despite many different techniques proposed in the literature, tracheal heterotopical allotransplantation still seems the most promising, and ALT seems to be a feasible alternative for heterotopical transplantation of trachea.
